# Acute fatal ventricular arrhythmia induced by severe hyperkalemia in a toddler with decompensated methylmalonic acidemia

**DOI:** 10.1186/s13256-024-04406-5

**Published:** 2024-02-24

**Authors:** Zahra Hakimzadeh, Abolfazl Gilani, Parsa Yousefichaijan, Roham Sarmadian

**Affiliations:** 1 Faculty of Medicine, Tabriz University of Medical Sciences, Tabriz, Iran; 2 Department of Pediatric Surgery, Tehran University of Medical Sciences, Tehran, Iran; 3 Department of Pediatrics, Arak University of Medical Sciences, Arak, Iran; 4 Infectious Disease Research Center, Arak University of Medical Sciences, Arak, Iran

**Keywords:** Methylmalonic academia, Hyperkalemia, Ventricular tachycardia, Genetic disorders

## Abstract

**Background:**

Methylmalonic acidemia is a very rare genetic metabolic disease. Patients with isolated methylmalonic acidemia typically present with acute alterations of consciousness, failure to thrive, anorexia, vomiting, respiratory distress, and muscular hypotonia. Despite the evidence-based management, affected individuals experience significant morbidity and mortality. Hyperkalemia is one of the unusual complications of methylmalonic acidemia.

**Case presentation:**

In this paper, we describe a 4-year-old Persian boy with methylmalonic acidemia who developed life-threatening arrhythmia following severe hyperkalemia and metabolic acidosis. Emergent management of the condition was successfully carried out, and the rhythm changed to normal sinus rhythm by effectively reducing the serum potassium level. We discuss the possible etiology of this lethal condition and describe its management on the basis of the available evidence.

**Conclusion:**

During metabolic decompensation in methylmalonic acidemia, frequent blood gas and electrolyte testing to prescribe and adjust therapy and annual echocardiogram and electrocardiogram screening are essential.

## Introduction

**Fig. 1:**
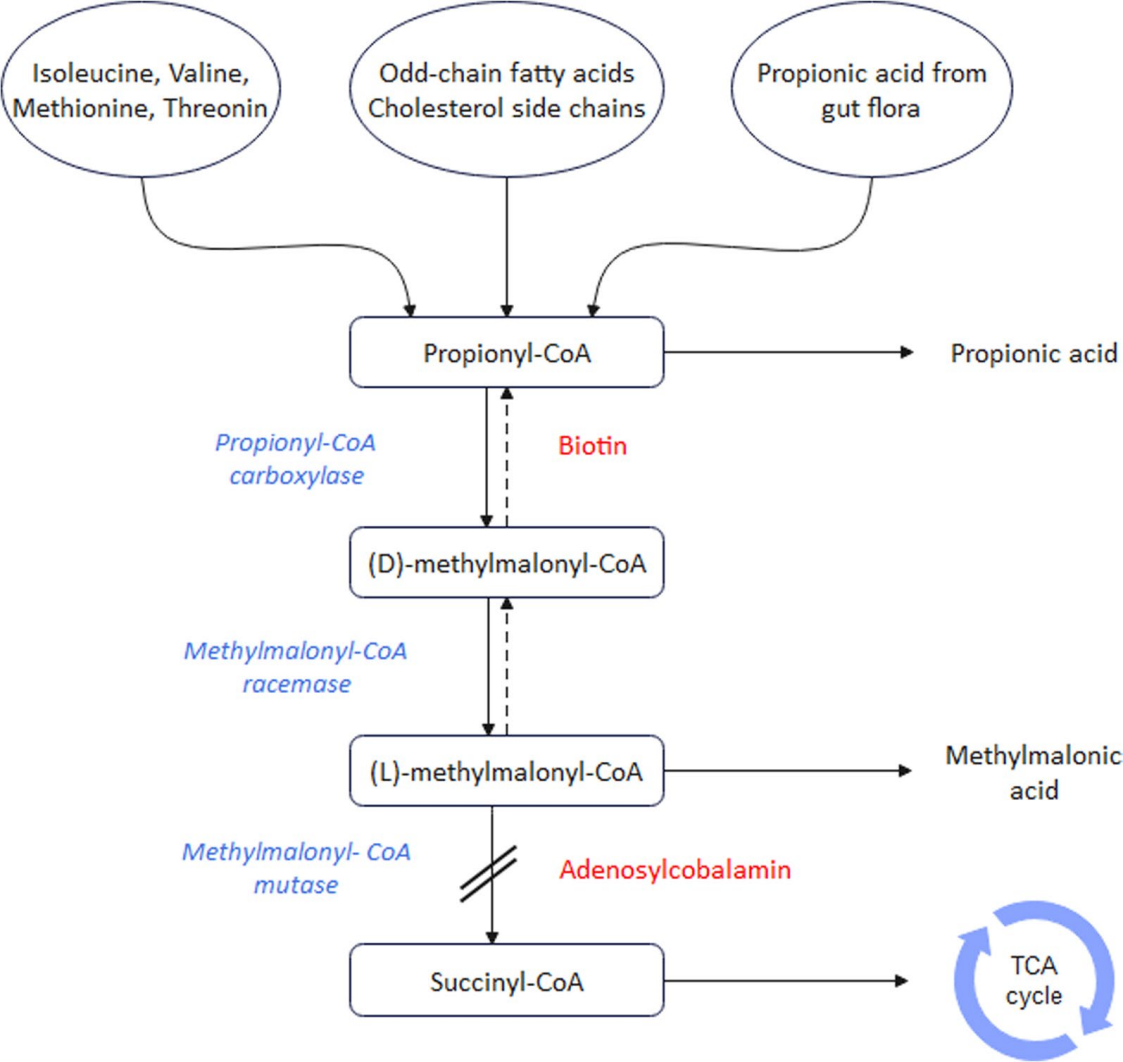
The metabolic pathway of methylmalonic acidemia (MMA). The accumulation of methylmalonic acid occurs owing to defects in either intracellular methylmalonyl-CoA mutase (MCM) or adenosylcobalamin, which serves as the coenzyme for MCM

Methylmalonic acidemia is a rare autosomal recessive inborn amino acid metabolism disorder, associated with a defect in the conversion of methylmalonyl coenzyme A (CoA) to succinyl CoA [1]. Isolated methylmalonic acidemia (MMA) is caused by an extreme deficiency or a lack of activity of the methylmalonyl-CoA mutase (MCM) enzyme, which needs adenosylcobalamin (AdoCbl) as a cofactor, whereas combined methylmalonic acidemia and homocystinuria is caused by elevated plasma homocysteine and decreased levels of the coenzymes AdoCbl and methylcobalamin (MeCbl; Fig. 1) [2].

Depending on genetic inheritance, MMA symptoms can appear at any time between the neonatal period and later in childhood [3]. MMA has a wide clinical spectrum, ranging from benign conditions to a lethal neonatal disease [4]. Patients with isolated MMA commonly present with acute alterations of consciousness, failure to thrive, poor appetite, frequent vomiting, dehydration, muscular hypotonia, and respiratory distress [4–6].

Long-term complications of MMA include growth retardation, neurological abnormalities (basal ganglia lesions), dystonia and para/quadriparesis, functional immunodeficiency, pancreatitis, optic nerve atrophy, and tubulointerstitial nephritis with progressive renal failure [2, 7, 8]. End organ damage happens as a result of primary toxicity of the accumulating metabolites as well as deficiency of succinyl-CoA leading to Krebs cycle and oxidative phosphorylation dysfunction [9].

Laboratory analysis shows metabolic acidosis, ketonemia or ketonuria, lactic acidosis, hyperammonemia, hypoglycemia, leukopenia, thrombocytopenia, anemia, and C3 acylcarnitines and organic acid elevation in the urine [2, 10]. The high concentrations of methylmalonic acid and methyl citrate in urine can result in conclusive diagnosis of the disorder [2]. The pattern of renal manifestations includes defects in urine concentration and acidification due to tubular damage, and progressive impairment of renal function due to chronic interstitial nephritis [11]. Hyperkalemia is a rare manifestation of MMA, and to our knowledge, only one case of severe hyperkalemia leading to life-threatening cardiac arrhythmia has been described in the literature [12].

Hyperkalemia after acute metabolic decompensation can occur even when renal function is normal [12, 13].

Herein, we report a rare case of MMA presenting with ominous arrhythmia following severe hyperkalemia and metabolic acidosis and explain the successful management of this lethal condition.

## Case report

A 4-year-old Persian boy, 10 kg in weight and diagnosed with MMA, was referred and admitted to our hospital because of progressive lethargy after frequent vomiting since a day before hospitalization and excessive crying and irritability, upward gaze, and muscular hypotonia on the day of hospitalization.

He was the second child of healthy, related parents (cousins), born after an uneventful pregnancy and delivery with a birth weight of 2,750 g. His older brother was healthy. His family had a low-class income. His father works at a university and his mother is a housewife. The diagnosis of MMA was made at the age of 3 months on the basis of the clinical presentation and series of laboratory tests (metabolic acidosis, high level of ammonia, and high urinary concentration of methylmalonic acid). The patient had a history of frequent hospitalization owing to imbalanced electrolyte levels and he was regularly taking prescribed medications for 6 months including oral solution of cyanocobalamin 1000 mcg daily, l-carnitine (100 mg/kg/day), Shohl’s solution (sodium citrate) 30 ml every 6 hours.

**Fig. 2:**
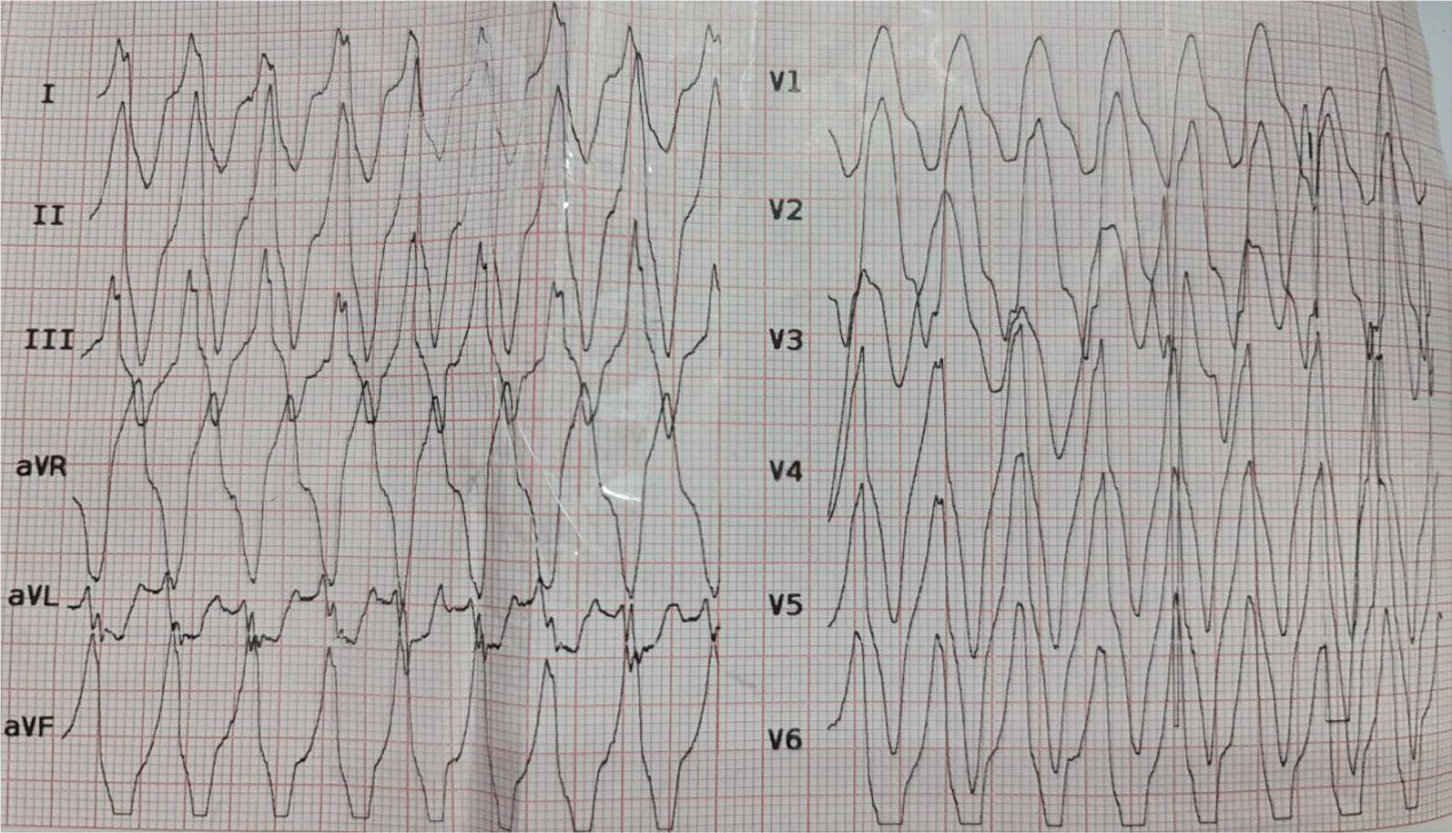
Ventricular tachycardia caused by hyperkalemia

Upon the patient’s arrival in the emergency room, owing to abnormal vital signs [temperature (T): 36.3 °C, pulse rate (PR): 280, respiratory rate (RR): 45, blood pressure (BP): 100/90 mmHg, and O_2_ saturation: 97%]. Electrocardiography (ECG) was performed and showed wide QRS complex ventricular tachycardia (Fig. 2).Physical examination revealed pale skin, lethargy, and upward gaze. The muscular examination revealed hypotonia. Neurological examination: all cranial and peripheral nerve examinations were normal. During the present episode, the patient’s laboratory test results were as follows: red blood cell (RBC), 3,530,000/µl; white blood cell (WBC), 8210/µl; hemoglobin (Hb), 9.2 gr/dl; platelet (plt), 375,000/µl; alanine transaminase (ALT), 13; aspartate aminotransferase (AST), 24; alkaline phosphatase (ALP), 499; hepatitis B surface antigen (HBSAg), negative; and human immunodeficiency virus antibody (HIVAb), negative. Urine analysis: WBC, 0–1; RBC, 0; epithelial cell, 1–2; crystals, not seen; cast, not seen; and bacteria, not seen. Arterial blood gases (ABG) revealed severe metabolic acidosis (pH = 7.13, HCO_3_ = 9.5 mmol/L, pCO_2_ = 28.1 mmHg), hyperkalemia (*K* =  8.9 mEq/L, Na = 136 mEq/L), and renal insufficiency (serum creatine levels = 2.3 mg/dl and urea nitrogen = 80 mg/dl). Serum ammonium and lactate level was 95 mcg/dl and 26 mg/dl, respectively. Severe hyperkalemia and metabolic acidosis with respiratory compensation were diagnosed.

The patient immediately underwent treatment, including calcium gluconate 10% intravenously infused over 10 minutes, sodium bicarbonate direct intravenous injection (2 mEq/kg) over 5 minutes, hydration with dextrose 10% intravenous serum, and regular insulin intravenous infusion (0.1 ml/kg/hour), and was transferred to the pediatric intensive care unit (PICU) for further assessments. His blood pressure (BP) was 100/70 mmHg, pulse rate was 180/minute and irregular, respiratory rate was 42/minute, body temperature recorded 35.9 °C by skin, pulse oximetry indicated 97% in the PICU. The frequency of ventricular tachycardia gradually decreased and the ECG showed sinus tachycardia. Repeated ABG showed improvement (pH = 7.43, HCO3 = 13.3 mmol/L, pCO2 = 21 mmHg), and serum potassium level came down to 6 mEq/L within the next 6 hours. This management was continued under the observation of a multidisciplinary team. Additionally, kayexalate (10 g every 6 hours) as a sodium potassium exchange resin therapy and MMA formula were added to the treatment routine for the next 2 days. The patient stabilized, serum potassium level reduced to 4.9 mEq/L, serum creatine level reduced to 2.1 mg/dl, and urea nitrogen reduced to 57 mg/dl. It is notable that brain magnetic resonance imaging (MRI) was normal and no growth was observed in patient’s blood culture. He was discharged with a good clinical condition. During the past 6 months, the patient has been regularly monitored every 2 months and has not exhibited any new neurological, cardiological, or other issues. Follow-up is ongoing.

## Discussion

The current MMA case depicts a rare instance of severe hyperkalemia-induced life-threatening ventricular tachycardia. This uncommon case, characterized by metabolic acidosis, renal failure, and prompt response to a multimodal treatment approach, offers useful insights into the management of a critical condition in pediatric patients with MMA.

MMA is a rare inborn error of amino acid metabolism, which leads to significant morbidity and mortality. Standard management of the disease includes low-protein high-energy diet, l-carnitine, vitamin B12 in responsive patients with MMA, mineral supplementation, and metronidazole (to reduce the burden of propiogenic gut flora) [3, 14].

Despite best-practice management, these patients are at increased risk for acute metabolic decompensation during mild viral illnesses or other emotionally and physically stressful events that may cause catabolism [9]. These decompensation events usually manifest as anorexia or enteral feeding intolerance, vomiting, and altered consciousness or lethargy with laboratory investigations indicating metabolic acidosis and electrolyte imbalance, as seen in our case [15].

Management of the metabolic decompensation of MMA includes intravenous fluids containing dextrose (typically 10% with appropriate electrolyte additives) to prevent protein and fat catabolism, associated with insulin infusion to promote anabolism while maintaining normoglycemia [3]. Solid organ transplantation can protect patients against acute metabolic decompensation but has numerous practical limitations [2, 16].

Several hypotheses and pathophysiologies can be proposed regarding the cause of hyperkalemia in our patient. Firstly, the accumulation of methylmalonic acid can cause metabolic acidosis; therefore, to correct the acidosis, the body pulls hydrogen ions into the cells by exchanging them with potassium ions. This can cause potassium to move out of cells into the bloodstream, causing hyperkalemia [17]. Secondly, MMA can cause renal dysfunction, resulting in decreased potassium excretion, leading to hyperkalemia [18, 19]. Thirdly, in severe MMA cases, there can be major tissue damage. This releases potassium into the bloodstream, causing hyperkalemia [20]. Finally, dehydration and renal hypoperfusion, preexisting renal disease, hypercatabolism, and therapy courses may contribute to hyperkalemia in patients with organic acidemias [21].

Hyperkalemia has depolarizing effects on the heart that are revealed by progressive changes in the ECG including: peaked T waves (tall, narrow, and symmetric), ST-segment depression, widening of the PR interval, widening of the QRS complexes, loss of the P wave and sine wave pattern (indicating impending ventricular fibrillation), and asystole [22, 23].

To treat this condition, intravenous calcium (rapidly normalizes membrane excitability) and insulin (promote potassium entry into cells) are beneficial. To prevent hypoglycemia, short-acting insulin should be accompanied by glucose infusion. Sodium bicarbonate should be used in patients who are hyperkalemic with metabolic acidosis after receiving insulin and glucose [22].

Recently, Chao *et al*. reported a case of MMA with wide QRS complex ventricular tachycardia and hyperkalemia following induction of anesthesia. They reported that, in spite of using lidocaine as a antiarrhythmic to suppress sustained ventricular arrhythmia, the rhythm changed to low voltage electrical activity [12]. Moreover, previous studies have suggested that antiarrhythmic therapy is less effective in the presence of severe hyperkalemia [24]. Therefore, serum potassium concentration should be reduced directly insisted of following the wide QRS complex ventricular tachycardia treatment algorithm [25].

## Conclusion

In the metabolic decompensation phase in methylmalonic acidemia, frequent evaluation of blood gas and electrolytes to prescribe and change therapy according to needs and annual screening with echocardiography and ECG are crucial [9, 13].

## Data Availability

Not applicable.

## Abbreviations

MMA: Methylmalonic academia
CoA: Coenzyme A
AdoCbl: Adenosylcobalamin
MeCbl: Methylcobalamin
BP: Blood pressure
PICU: Pediatric intensive care unit
ABG: Arterial blood gases
ECG: Electrocardiography
